# Human Brain Organoids as an *In Vitro* Model System of Viral Infectious Diseases

**DOI:** 10.3389/fimmu.2021.792316

**Published:** 2022-01-11

**Authors:** Xuan Su, Peng Yue, Jing Kong, Xin Xu, Yu Zhang, Wenjing Cao, Yuxin Fan, Meixiao Liu, Jingjing Chen, Aihua Liu, Fukai Bao

**Affiliations:** ^1^ Yunnan Province Key Laboratory for Tropical Infectious Diseases in Universities, Kunming Medical University, Kunming, China; ^2^ Department of Pediatrics, The Affiliated Children Hospital, Kunming Medical University, Kunming, China; ^3^ Department of Biochemistry and Molecular Biology, Kunming Medical University, Kunming, China; ^4^ Department of Microbiology and Immunology, Kunming Medical University, Kunming, China

**Keywords:** brain organoid, brainoid, infection, virus, prion, model system, pathogenesis

## Abstract

Brain organoids, or brainoids, have shown great promise in the study of central nervous system (CNS) infection. Modeling Zika virus (ZIKV) infection in brain organoids may help elucidate the relationship between ZIKV infection and microcephaly. Brain organoids have been used to study the pathogenesis of SARS-CoV-2, human immunodeficiency virus (HIV), HSV-1, and other viral infections of the CNS. In this review, we summarize the advances in the development of viral infection models in brain organoids and their potential application for exploring mechanisms of viral infections of the CNS and in new drug development. The existing limitations are further discussed and the prospects for the development and application of brain organs are prospected.

## Introduction

The study of human viral infections is limited by the lack of functional models that simulate normal human physiology and pathophysiology (1). For decades, human pathogenic viruses have been studied using immortalized cell lines, primary cells isolated from the body, and a variety of animal hosts (2). Although these traditional models have made significant progress in helping to understand viral pathogenesis and host pathogen interactions, and have contributed to the development of vaccines and treatment strategies, these models may have limitations in reproducing interactions between pathogens and human hosts (1). Recently developed humanized mouse models are more valuable for human viral disease research, but they are expensive and difficult to maintain (3). A two-dimensional (2D) research strategy based on induced pluripotent stem cells (iPSC) has provided valuable information for the pathophysiology of neurological diseases, but lacks three-dimensional (3D) properties of their internal structures; thus, tissue structures composed of many different types of cells, and their complex environments and functions cannot be simulated (1, 3, 4). Although the 3D-based organoid culture research strategy is still in its infancy, it has provided a new method for modeling human disease **(** **Table 1** **)** (4). Organoids are derived from human stem cells and retain the genomic background, cellular organization, and function of their tissues of origin. Recent advances in stem cell biology have made it possible to grow organoids *in vitro*, mimicking a variety of human tissues, including the brain, lungs, and intestines (2).

**Table 1:** Advantages and disadvantages of the *in vitro* models used in the study of viral diseases in the CNS.

In vitro models	Types	Advantages	Disadvantages
Cells	primary cells	Morphologically and physiologically similar to human neurons. Similar to the proliferation rate of human neurons. Suitable for generation of genetic model. Cells are widely available, inexpensive and can be cultured in large numbers.	Ethical problems, mixed cell culture, variations among different culture preparations and difficult to maintain. Dissection needed, procedure can introduce experimental variability. Species-dependent cell differences.
	cell lines	Easy to culture and transfect. Easy to maintain and store. Homogenous populations, suitable for large-scale experiments. Cell lines often are immortal and can be passaged for long time. Study results are easy to be comparable among different laboratories.	Lacks native cell phenotypes. High-number passages may lead to genetic phenotype changes. Tumor-derived or virus-transformed cell origins may influence cell differentiation, metabolic properties, viability, or growth performance.
	iPSCs	Self-renewal ability is strong. Allows the generation of autologous pluripotent cells from any individual for disease modeling. The potential to differentiate into any cell type. Suitable for research and personalized medicine.	Challenging to identify disease-specific cell phenotypes that better represent pathogenesis. Requirements for standardized protocols and quality control to minimize technical changes. High costs.
Organoids	regular organoids	Confers a three-dimensional organization closer to human tissue, providing the cells with apical-basal polarity and cellular interactions, resembling *in vivo.* Microenvironments proper for virus–host interactions. Simulate native cell–cell communication and cell–ECM interaction. Promising for screening compounds targeting the central nervous system	Highly variable. Without blood vessels, all cells cannot be properly nourished. Time consuming, expensive. Ethical problems.
	organoid-on-a-chips	The organ-on-a-chip systems allow the creation of dynamic and controllable microenvironments proper for viral infections and immune response analysis. With microfluidic devices used in organ-on-a-chip, it is possible to simulate human vascular endothelial dynamics. The organoid-on-a-chip can evaluate interactions with cells of the immune system and the cellular response to viral infections.	A single organ on a chip has limited capacity to meet the wide range of needs emerging from drug discovery. Non-adhesive culture conditions and cell interactions are difficult to achieve in microfluidic chips. As far as genomic stability is concerned, organ-on-a-chip systems cannot fully recapitulate cell types and their ratios *in vivo* and may introduce unexpected mutations during cultivation.

A growing body of clinical data suggests that many pathogen infections eventually lead to a range of brain diseases. The development of the human brain presents several unique aspects, such as higher complexity and the expansion of neuronal output, which have proven difficult to study using traditional biological models (5). Thus, developing treatments for brain diseases is challenging because it is difficult to translate preclinical data from current *in vitro* and *in vivo* models into clinical practice (6). As a result, *in vitro* methods to simulate human brain development and disease have become a hot research area (2). As early as 1907, Harrison took frog nerve tubes and attached tissue fragments to a glass cap of coagulated serum or lymph, and thus established a hanging drop culture that allowed direct observation of growing nerves while still alive (7). In 2001, researchers produced the first 2D embryonic stem cells (ESCs) to simulate the development of embryonic neural tubes (8). In 2008, scientists modified ESCs to differentiate into dorsal forebrain progenitor cells, mimicking the main pattern of *in vivo* cortical development (9). Later, Pasca et al. generated pyramidal neurons from hiPSCs in three-dimensional cortical structures called human cortical globules (hCSs) (10). In 2013, Lancaster et al. combined the three-dimensional matrix and serum-free growth techniques when culturing iPSCs to generate a model of various brain regions with single-tissue methods (11).

**Figure 1:**
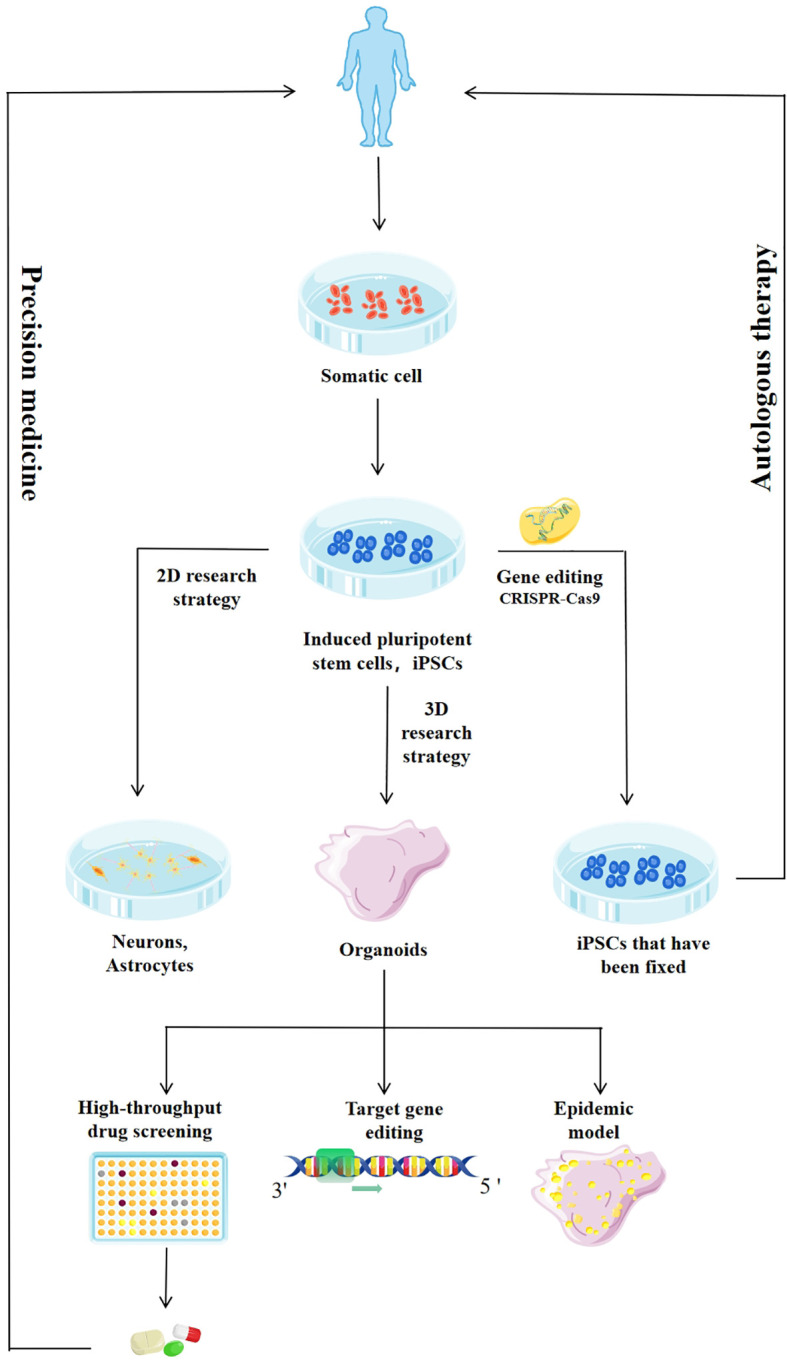
Application of induced pluripotent stem cells (iPSCs) and iPSCs-derived system. The enhanced culture and differentiation of iPSCs will improve the efficiency and quality of the organoids derived from iPSCs. The use of gene editing techniques such as CRISPR-Cas9 and specific small molecules will allow the generation of terminally differentiated patient cells and isogenic lines, reducing background genetic variation, and broadening the range of cells available for drug screening. Targeted differentiation schemes can be used to generate neural progenitor cells, which can be further induced into specific populations of neurons or glial cells. Two-dimensional (2D) cultures produce highly enriched cell populations to study the role of drugs in specific cell types. Three-dimensional (3D) cultures produce organoids that are similar in organization and function to the developing human brain and are used in high-throughput drug screening, genetic research, and infectious disease modeling, and to drive the development of precision medicine.

Furthermore, researchers achieved neural induction by high-density monolayer cultures or by embedding hiPSC clusters in gel-protein mixtures, such as Matrigel, and then cultured them in a rotating bioreactor, to mimic the 3D cortical-like structure of the brain, and produced pyramidal neurons (10). Recent advances in human iPSCs-derived brain-like organs offer promising approaches to the study of neurodevelopment, infectious diseases, high-throughput drug screening, and the genetic basis of various neuropathologies, and promote the application of precision medicine (12). Furthermore, the use of CRISPR-Cas9 gene editing techniques to reprogram iPSCs derived from patient somatic cells has brought new prospects to many areas of neurobiology, providing a wider range of options for culture systems. In theory, individualized therapy for many diseases is possible **(** **Figure 1** **)** (12, 13). Therefore, brain organs, or brainoids do provide a good opportunity for the study of brain diseases. But one limitation in these systems is that they can’t build all the types of cells that make up the central nervous system. For example, there are astrocytes, oligodendrocytes, neurons, ependyma and microglia cells, but no immune cell and vascular structure in these systems. In this review, we discussed the use of brain organoids derived from human stem cells in the study of various viruses, such as Zika virus (ZIKV), SARS-CoV-2, and human immunodeficiency virus (HIV), as well as prion infections.

## Brain Organoids as a Model of ZIKV Infection

ZIKV is an enveloped RNA virus of the Flaviridae family, the ZIKV genome contains one open reading frame (ORF) and 5′ and 3′ untranslated regions. The single polyprotein encoded the ORF can be further cleaved into ten functional proteins, including three structural proteins (as components of envelope, precursor membrane, and capsid) and seven nonstructural proteins (NS1, NS2A, NS2B, NS3, NS4A, NS4B, and NS5) (14). Mainly transmitted by *Aedes aegypti*, ZIKV was first discovered in the blood of an outpost rhesus monkey in Uganda in 1947. But ZIKV caused its first major epidemic in Micronesia, with patients developing fever, joint pain, rashes and conjunctivitis until 2007. ZIKV was transmitted to Brazil in 2013 and found to be associated with microcephaly (15–18). There is also evidence that ZIKV can be transmitted sexually and from mother-to-child, and that ZIKV infection in pregnant women increases the prevalence of microcephaly and other neurodevelopmental disorders in newborns (19). Computed tomography images of several infants infected with ZIKV revealed intracranial calcification, severe brain atrophy, and cerebellar hypoplasia (20). In December 2016, the World Health Organization (WHO) declared ZIKV infection a public health emergency. However, the pathogenesis of ZIKV infection, how it affects neurons, and the efficacy of drugs have not been fully understood until recently, and the study of ZIKV in brain organoids has contributed to the rapid progress in elucidating the pathogenesis of ZIKV.

Several studies have shown that ZIKV infection can reduce the growth of organoids by increasing the death and proliferation of developing neurons, this leads to a reduction in the volume of the neuronal cell layer, which disrupts the formation of the neurospheres and in turn, reduces the growth of organoids (21, 22). Another study found that morphological changes of human neural progenitor cells (hNPCs) apparently took place and the fluorescence signal of dead cells was signifificantly increased in NS5-expressing hNPCs, consistent with ZIKV infection, ZIKV-NS5 could induce apoptotic cell death of hNPCs through regulating p53 activity. Interestingly, the expression of ZIKV-NS5 alone in hNPCs could induce the p53-mediated apoptosis, implying its contribution to the microcephaly caused by ZIKV infection (14). Their results demonstrated that ZIKV induced cell death in human iPS-derived NSCs, disrupted the formation of neurospheres, and reduced the growth of organoids (17). Fetal ZIKV infection has been identified as the cause of brain defects such as microcephaly, but live infected human fetal tissue is not available and postmortem tissue is of different quality and genetic background; clinical studies alone cannot provide sufficient insight into how ZIKV causes this damage (21). Using a developmental brain model based on ESCs, researchers studied the effects of ZIKV infection on patterns of DNA methylation throughout the genome of selected nerve cell types. ZIKV unexpectedly altered the DNA methylation status of neural progenitor cells, astrocytes, and differentiated neuron genes; up to 10% of these genes are associated with susceptibility to schizophrenia, bipolar disorder, or other diseases (23). The findings suggest that microcephaly and other large brain abnormalities in babies born after ZIKV infection during pregnancy may be just the beginning, there may also be a range of neuropsychiatric complications associated with delayed onset (23). The brain organoids are valuable in elucidating the mechanisms behind these phenomena. To understand how the virus travels through the blood to mature endothelial cells and to tissues, the researchers used primary human mononuclear cells, brain-like organoids from ESCs, slices of the cerebellum of organ-type mice, xenogenetic zebrafish models and human fetal brain samples, and found that ZIKV-infected monocytes showed higher expression of adhesion molecules, and ZIKV induced human primary monocytes to have higher adhesion, diffusion, and migration in a blood-brain barrier (BBB)-like model *in vitro* (24). Based on transcriptomic analysis, ZIKV infection resulted in an up-regulation of Toll-like receptor 3 (TLR3) expression that derailed cell fate and reduced the number of functional neurons, leading to microcephaly (17, 25). At the molecular level, Yoon et al. found that the ZIKV-encoded NS2A protein disrupted the proliferation of radial glial cells in both the embryonic mouse cortex and the human forebrain, and ZIKV-NS2A disrupted the formation of the adhesive junction complex, resulting in aberrations of niche signals affecting cortical neurogenesis (26). Furthermore, because of the high expression of receptor tyrosine kinases in human radial glial cells (RGCs) and outer radial glial cells (oRGCs), AXL is thought to be the receptor protein for ZIKV entry in neural progenitor cells (NPCs) (21). Another study in 2016 found that ZIKV AXL receptors were highly concentrated in the radial glia of NSCs in the human embryonic cerebral cortex, providing a hypothesis that these cells are particularly vulnerable to ZIKV infection, it provides a candidate mechanism for ZIKV virus to induce microcephaly (27). A study showed that the RNAi mechanism in the hNPCs was fully capable of cleaving the dsRNAs formed during the replication of the ZIKV genome, demonstrating the direct antiviral activity of enoxacin, a well-known RNAi enhancer, and enoxacin treatment can completely prevent ZIKV induced microcephaly in brain organoids (28). These studies showed that brain organoids are of great value in studying the effects of ZIKV at the structural, cellular, and genetic levels. Brain organoids infected with ZIKV are also used in high-throughput drug screening. The researchers screened about 6000 compounds for drug reuse and found that treatment with emricasan and clonitazene restored ZIKV-negative hNPCs. It is suggested that infected cells could recover by inhibiting apoptosis to gain time (29). High-level chemical screening of cortical hNPCs derived from human multipotent stem cells showed that hippeastrine hydrobromide and amodiaquine dihydrate (AQ) could inhibit ZIKV infection (30). The methylene blue (MB), approved by the United States Food and Drug Administration (FDA), inhibits the interaction between viral protease NS3 and its cofactor NS2B, inhibits viral protease activity and viral growth, and protects 3D cerebellar organoids from ZIKV infection. Mechanistic studies have confirmed that MB plays a role in ZIKV infection and post-infection, with MB most effective for ZIKV two hours before infection (31). Furthermore, bioinformatics analysis showed that betulinic acid (BA) treatment activated the AKT pathway, therefore, BA exerts a cell protective effect on neural progenitor cells infected by ZIKV (32). The broadly active antiviral compound TH6744 interferes with the ZIKV life cycle of RNA replication, with the synthesis, posttreatment, or stability of viral proteins, and with the assembly, transport, or budding of viral particles in the brain organoid model. Therefore, TH6744 can not only alleviate the pathogenic phenotype induced by ZIKV, but also blocks the transmission of the virus (33).

## Brain Organoids as SARS-CoV-2 Infection Model

According to national authorities, as of 11 October 2020, severe acute respiratory syndrome coronavirus 2 (SARS-CoV-2), the causative agent of the 2019 coronavirus pandemic, was responsible for more than 37 million confirmed cases of COVID-19 and 1 million deaths (34, 35). SARS-CoV-2 is a β coronavirus, derived primarily from bats and other species. The virus can cause respiratory and intestinal diseases in different animal species (36). Furthermore, clinical observations revealed neurologic symptoms and neuropsychiatric disorders in patients with COVID-19 (37). The latest clinical reports have shown that the number of patients with neurological symptoms continues to increase, and a considerable number of patients with cerebrovascular injury, emotional disorders, and other neurological complications have been reported (34, 38). On 30 January 2020, the WHO declared COVID-19 a public health emergency of international concern (39). Although the SARS-CoV-2 pathogen responsible for COVID-19 has been detected in the brains of some patients, the detailed mechanisms through which it infects brain cells and affects their function are not known.

**Figure 2:**
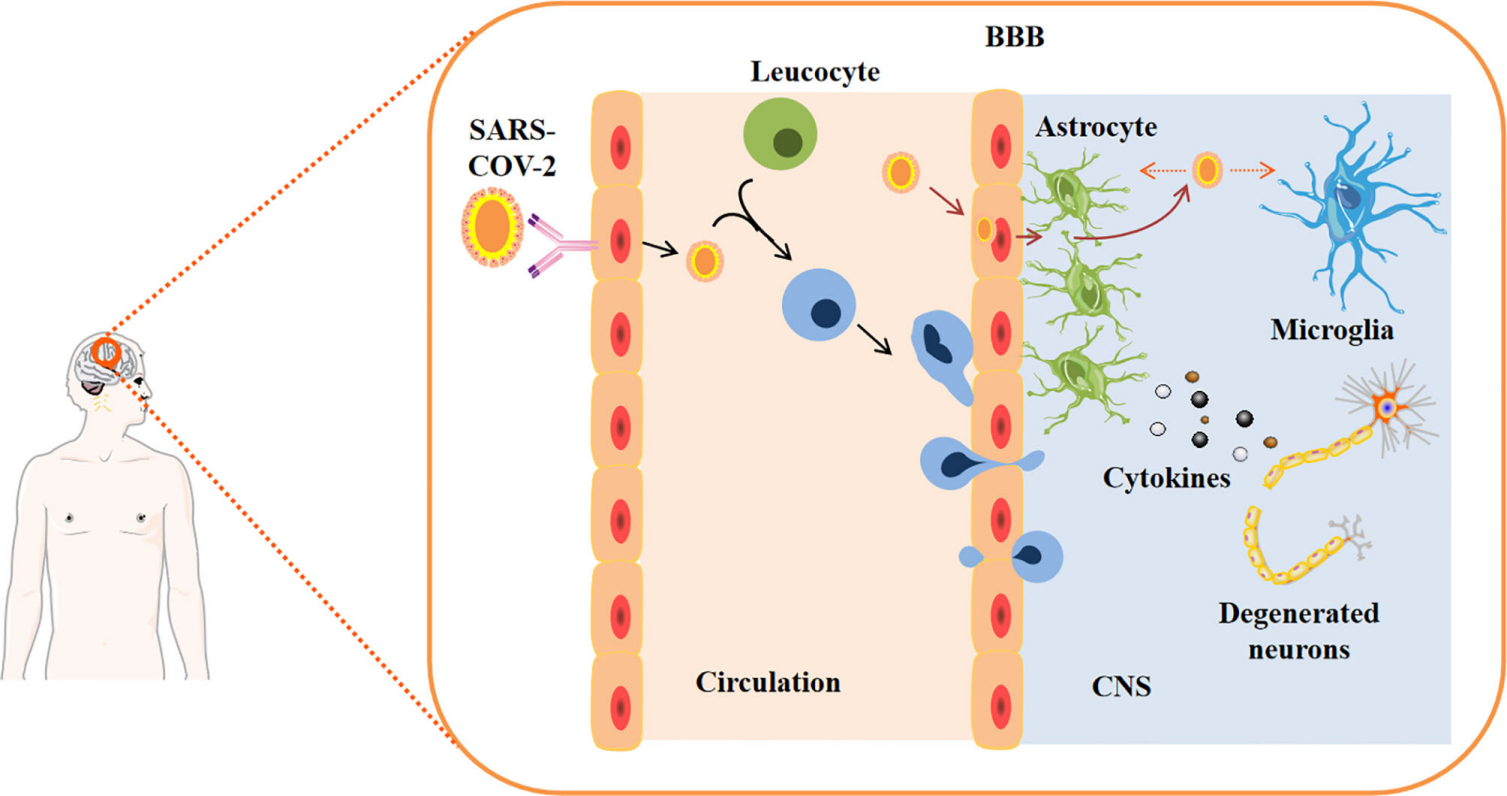
SARS-CoV-2 entry into the central nervous system. Viruses in the blood may infect peripheral immune cells. These infected white blood cells can cross the blood-brain barrier (BBB), which is made up of special tight junctions between endothelial cells, pericytes, and astrocytes. In addition, the virus may cross the BBB, which may be modified by cytokines, or enter the cerebrospinal fluid (CSF) through direct interaction with the endothelium. Both mechanisms lead to changes in brain homeostasis and increase the production of cytokines in the central nervous system.

To study the susceptibility to SARS-CoV-2 infection, researchers used single-layer brain cells and region-specific brain organoids derived from human iPSC as cell models, and found that a small number of neurons and astrocytes were infected; whereas, the epithelial cells of the choroid plexus can be heavily infected (34, 37, 38, 40, 41). The study also found that SARS-CoV-2 infection-induced neuronal apoptosis depends on the type II interferon response rather than on the type I interferon response (42). In another study, researchers challenged brain organoids with SARS-CoV-2 spinous pseudoviruses and live viruses, and also demonstrated viral propensity in choroidal plexus epithelial cells, but rarely or never infect neurons or glial cells (40). Researchers have discovered that viruses in the blood can infect peripheral immune cells. These infected white blood cells can pass through the BBB, which is made up of special tight junctions between endothelial cells, pericytes, and astrocytes. In addition, the virus may cross the BBB or enter the cerebrospinal fluid (CSF) through direct interaction with the endothelium. Both mechanisms lead to changes in brain homeostasis and increase the production of cytokines in the central nervous system (CNS) (43) **(** **Figure 2** **)**. Next, researchers studied the effect of SARS-CoV-2 infection on the function of choroid plexus (CHP) epithelial cells, and observed a sharp decrease in fluid accumulation in damaged organs, indicating a morphological disorder leading to the destruction of barrier integrity (37, 38, 40). The S1 subunit of SARS-CoV-2 was found to mediate barrier rupture in a 3D microfluidic model of the human BBB (44). At the molecular level, there was evidence of increased levels of Tau protein in the soma of SARS-CoV-2-positive neurons, and changes in the location of Tau and T231 phosphorylation of Tau protein in SARS-CoV-2-positive neurons. Therefore, the researchers concluded that the Tau protein is abnormally phosphorylated in response to viral-induced stress, which may in turn, trigger further cell death processes (38). Thus, brain organoids could model the pathologies affecting the CNS by COVID-19.

Researchers including Markus Hoffmann showed for the first time that Angiotensinogen 2 (ACE2) is the key receptor for SARS-CoV-2 entering cells (45). Several studies have shown that high levels of ACE2 are found in mature choroid plexus cells expressing rich lipoproteins, co-entry factors TMPRSS2 and TMPRSS4, with the abundance of ACE2 increasing over time, but it is less expressed in neurons and other cell types (38, 40, 43). An analysis of the data from Allen’s brain atlas revealed that the CHP had the highest level of ACE2, which confirmed the findings of an *in vitro* brain-like organ model (40). High expression of ACE2 in choroid plexus organs may explain their susceptibility to SARS-CoV-2 infection (35). However, some researchers still have doubts about whether ACE2 is the main pathway for SARS-CoV-2 entry into neuron cells. One reason is that the level of ACE2 mRNA in CNS seems to be very low. However, recent studies have shown that ACE2 mRNA levels are not the best substitute indicator of ACE2 protein expression (46). ACE2 protein is widely expressed in neurons positive for microtubule-associated protein 2 (MAP2) and in cells near the organoid lumen (46). Further, 2D monolayer cultures of cortical neurons and brain organoids were used to assess ACE2 expression and infectivity of the SARS-CoV-2 pseudovirus. The colocalization of the mCherry signal with Angiotensinogen-2 (ACE-2) from pseudovirus-infected cells indicated that the SARS-CoV-2 spike protein can target cells expressing ACE2 in brain organs (43). Therefore, a detailed study of the brain distribution of ACE-2 may shed light on potential SARS-CoV-2 induced neurological changes. Targeted drug inhibition of the ACE2 receptor may represent an opportunity to treat COVID-19. The researchers found that the FDA approved antiviral drug sofosbuvir could inhibit SARS-CoV-2 RNA-dependent RNA polymerase (RdRp) and prevent neuronal death (41). Other studies examining the inhibitory effects of remdesivir on SARS-CoV-2 infection in human iPSCs-derived brain cells, also support the anti-SARS-CoV-2 potential of remdesivir and provide evidence for its use in the treatment of neurological complications in patients with COVID-19 (47). This will bring new hope for the treatment of COVID-19.

## Brain Organoids as Models for HIV Infection

First isolated in 1983, the HIV is a retrovirus virus that causes acquired immune deficiency syndrome (AIDS) (48). The main modes of transmission of the virus are sexual transmission, blood transmission, and mother-to-child vertical transmission (49, 50). The brain manifestations of HIV infection with cognitive, behavioral, motor and autonomic dysfunction remain a problem in the daily management of HIV treatment. HIV infection may cause HIV-associated neurocognitive disorder (HAND) such as HIV encephalopathy and AIDS dementia (51). HIV is an infection caused by HIV-1 and HIV-2 lentiviruses, with HIV-1 infection rates much higher than HIV-2 (52). Infected individuals must, however, remain on antiretroviral therapy (ART) for the rest of their lives, because as soon as treatment is interrupted, viral loads return to pre-ART levels (53). Unfortunately, no clinically approved HIV-1 transcriptional inhibitors can block the ongoing transcriptional events in infected cells (53). In addition, HIV co-infection with *Mycobacterium tuberculosis* is a major public health concern worldwide, particularly in southern Africa (54). The genetic diversity of HIV-1 is striking, with more than 10 subtypes and an increasing number of recombinant strains around the world. However, it is not entirely clear whether subtypes have a biological effect on latency establishment and reversal. This complexity disturbs HIV vaccine development for which major epitopes of envelope proteins vary widely among different subtypes, thus affecting the efficacy of vaccines. To investigate HIV pathogenesis, researchers have developed a 3D human brain organ culture system (MG-hBORG) that adds HIV-infected microglia to human brain organ culture system (hBORGS), leading to inflammation and astrocyte damage, which is the main feature of the CNS damage (50). Furthermore, the study of brain cells reservoirs of HIV-1 that prevent a sterile cure is another interesting context in which brain organoids may be used in the future. But in the absence of suitable models, the pathogenesis of HIV-induced neurological disorders remains unclear. The generation of 3D brain organs provides a promising model system for studying the pathogenesis of HIV. Using CRISPR/Cas9 technology to edit HIV-1 proviral genes, Kunze et al. constructed an adeno-associated virus vector (AAV9P1), which demonstrated that AAV9P1 could provide tools for evaluating HIV-1 astrocyte infection. Inactivating the HIV-1 LTR, inhibits HIV-1 previral expression, with a subsequent decline in LTR activity induced by the LTR mutation, not the elimination of the original virus, so the development of the AAV vector, HIV suppression sequence to pass to the astrocyte, astrocyte may be an alternative strategy to control persistent HIV infection (49). Through the study of brain organoids, we will better understand the pathogenesis of HAND and HIV encephalitis (HIVE).

## Brain Organoids as a Model of Herpes Simplex Virus Infection

The human herpesvirus (HSV) is one of the most common human pathogens, and is highly prevalent worldwide and capable of forming lifelong infections. Although HSV infection is usually confined to the skin and mucous membranes of the lips and genitals, it is also a common cause of sporadic encephalitis, and can lead to potentially fatal CNS infection (55). The main causes of hospitalization in 106 patients with HSV encephalitis (HSVE), were seizures, abnormal behavior, loss of consciousness, confusion of consciousness, or disorientation. Second, weight loss and symptoms of infection, including low-grade fever and rash, as well as neurological or psychiatric disorders such as aphasia, behavioral changes, and epileptic activity, must also be reviewed (56). HSV-1 infection is human-specific and particularly common in adolescents and adults, and pregnant women are at risk of mother-to-child transmission, which can lead to fetal necrotizing encephalitis and even perinatal mortality (55). Sporadic encephalitis caused by HSV-1 infection affects 2-4 of 100,000 people annually, and although antiviral therapy with acyclovir derivatives significantly reduces mortality to about 25%, but sporadic encephalitis survivors often have serious and long-term neurological sequelae. Moreover, the pathophysiological characteristics of neurodevelopmental disorders associated with HSV-1 infection remain unclear (57). Therefore, it is necessary to study the genetics and epigenetics of HSV-1 and to establish an *in vitro* HSV-1 infection model of the human CNS to advance our understanding of the molecular mechanism of latency and reactivation of HSV-1. Alzheimer’s disease is a progressive neurodegeneration neuropathology characterized by the presence of extracellular amyloid plaques composed of amyloid beta (Aβ) peptides and intracellular neurofibrillary tangles. The scientists used human induced pluripotent stem cells (hiPSCs) to compare patterns of Ab42 accumulation in 2D (monolayer of neurons) and 3D neuron cultures (organoid brain) infected with HSV-1 (58, 59). Their *in vitro* models showed that hiPSC-derived CNS neurons allowed HSV-1 infection and that 2D neuron cultures showed Aβ42 immunoreactivity mainly in HSV-1-infected cells, rarely in uninfected cells or in infected cells that come in contact with antiviral drug. In contrast, 3D brain organoids showed that Aβ42 was mainly concentrated in uninfected cells surrounding HSV-1-infected cells. It is suggested that HSV-1 infection is a predisposing factor to the onset of Alzheimer’s disease. Further results showed that HSV-1 induced the accumulation of Aβ42 in monolayer culture of neurons, but antiviral therapy prevented it accumulation (58, 59). In another study, researchers generated *in vitro* models of neurodevelopmental disorders including single-layer neuronal differentiation of hiPSC, 3D shoots of neuroepithelial cells, and 3D brain organoids to study fetal brain development and potential effects of neuropathic HSV-1 infection, the results revealed that NSCs-infected with HSV-1 showed impaired neuronal differentiation, cortical and brain regionalization, and fetal neurodevelopmental disorder (55–57). Furthermore, it was emphasized that HSV-1 infection in the human brain showed a decrease in the thickness of cortical plate and a decrease in the expression of the LIM homeodomain transcription factor ISL1 (55). The 3D brain-like organ model also showed that HSV-1 infection can promote abnormal microglial activation and is accompanied by the induction of inflammatory factors such as Tumor necrosis factor (TNF)-α, interleukin (IL)-6, Il-10, and IL-4. HSV-1 infection inhibited cell population growth, at least in part by inducing apoptosis. The influence of HSV-1 infection on fetal brain development is highly dependent on the viral load of HSV-1 and the target cells (56).

In general, HSV-1 cannot be reactivated effectively, but it is known that it can be reactivated from peripheral neuron culture. HSV-1 is also difficult to reactivate due to latency in brain organoids, which is parallel to the inefficient reactivation of HSV-1 in the CNS (relative to peripheral nerves), therefore, brain organoids provide an effective model for studying the pathogenic mechanism of HSV-1 (57).

## Brain Organoids as Models of Prion Infection

Prion disease is a deadly neurodegenerative disease that has attracted widespread attention because of its transmissibility. Sporadic Creutzfeldt-Jakob disease (sCJD) is the most common prion disease in humans. Hereditary prion diseases are relatively rare and are associated with mutations in prion protein genes. To date, more than 50 different point mutations, deletions, and insertions have been identified. Most are autosomal dominant and completely permeable. Prion diseases also occur in animals and are of great concern due to their potential to be transmitted to humans (60). Prion diseases are usually sporadic, usually due to genetic mutations in the gene that encodes the prion protein or to exposure to prion-contaminated material. Different molecular subtypes of prions have been found to influence the clinical and pathological phenotypes in sCJD. Pathological features of prion diseases include loss of neurons, activation of microglia and astrocytes, spongy changes, and aggregation of pruritic prion proteins. However, the pathogenesis of prion diseases is not fully understood and diagnosis is possible only when the disease has progressed to intermediate or advanced stages (61).

iPCS-derived human brain organoids can be used to simulate prion transmission and reproduction, providing a powerful research tool to study the pathology of human prion diseases caused by different subtypes and as cell models to test treatments (61, 62). iPSC models derived from patients with Gerstmann-Sträussler-Scheinker syndrome (GSS) were used to study the molecular mechanisms involved in prion disease. A detailed analysis of immunoreactive cells showed that at the later stages of the human prion infection, proteins harboring the pathogenic mutation Y218N in culture, induced hypertrophic reactive astrocyte cells containing high levels of glial fibrillary acid protein and formed thick bundles. Finally, the nuclear staining analysis of the differentiation culture showed that chromatin condensation and apoptosis increased in cases with Y218N mutants. In order to replicate the human prion infection and pathogenesis, the researchers inoculated brain tissue homogenates of patients with different subtypes of spontaneous CJD to brain organoids, which revealed that the amount of prions invading brain organoids was influenced by the spontaneous CJD subtypes (62). The researchers also identified human APOE E4 as a risk factor for CJD (63). Furthermore, the researchers found that prions are transmitted primarily vertically, but studies in brain organoids have shown that prions can also be transmitted horizontally through vesicles without cell-to-cell contact (64). Therefore, brain organoids are an ideal model for studying the pathogenesis and transmission of prion.

## Brain Organoids as a Model for Human Cytomegalovirus Infections

Human cytomegalovirus virus (HCMV) infection is one of the main causes of morbidity and mortality in immunodeficient patients and the most common viral infection in developing human fetuses. Most HCMV infections are asymptomatic; however, when primary (and sometimes non-primary) HCMV infections occur in pregnant women, the virus can cause damage to the CNS (65–67). Thus, the development of the HCMV vaccine is considered a public health priority (66). However, due to the uniqueness of the epidemiology of HCMV infection in mothers, the general strategy of vaccine development is limited (67). It is particularly important to find an effective model for vaccine development. One study showed that the HCMV strain TB40/E can infect human brain organoids and induce growth and structural abnormality of human brain organoids. A neutralizing antibody targeting HCMV pentamer complex (PC) epitope can effectively prevent the infection of human brain organoids, and thus ensure the normal growth of human brain organoids and the formation of cortex. Further study showed that HCMV infection in human brain is related to platelet-derived growth factor receptor (PDGFR) and epidermal growth factor receptor (EGFR), and it does not seem to be dependent on integrins such as integrin α3, α5, or β3 (65). Thus, the use of brain organoids offers new hope for investigating pathogenic mechanisms and developing a vaccine for HCMV.

## Discussion

### Challenges and Future Perspectives

Although brain organoids offer new hope for studying human neurological disorders, they are still in their infancy and still have many limitations. First, more than 2 days of cell culture or self-organized neural rosette formation is required, which adds to the cost of each culture, in addition to the specialized and complex culture conditions, thus model requirements are higher (1, 68). Second, in the absence of an embryonic axis to guide the development of the fetal brain, as in other neural cultures, brain organoids cannot mimic the overall shape of a developing human brain. The heterogeneity and inconsistency of brain tissue cultures *in vitro* limit its reproducibility in the study of disease pathogenesis (68). Third, current brain organoids lack the components of a normal host microenvironment, such as immune cells and blood vessels. Due to the absence of immune components in brain organoids, it is difficult to reproduce a complex immune response and inflammatory processes following viral infection. Because brain organs do not have a vascular system, the size of the growing brain organs will be limited to 5-10 mm and may not integrate effectively with host tissues (1, 67).

Despite these challenges, the brain organoid, or brainoid, model represents an innovative and effective *in vitro* approach that encapsulates key features of a normal brain *in vivo* that many conventional cell and animal studies have proven difficult to achieve, and also replicates the pathological processes that mimic neurological disorders at gross morphological, protein, and genetic levels (68, 69). Genetic manipulation of brain organoids could be accomplished by stable editing of the hPSC genome or by direct infection of brain organoids by electroporation or retrovirus-associated viruses. Therefore, it is possible to study how individuals or combinations of ZIKV-encoding proteins or noncoding RNAs derived from ZIKV affect the development of brain-like organs (70). Furthermore, co-infection with HCV-HIV or co-infection with HIV-*Mycobacterium tuberculosis* are very common in the clinic, although it remains to be studied whether co-infection has a more severe impact on the CNS; in this context, the brain organoids represent an effective model to investigate the effects of these co-infections (3, 54).

The combination of human-specific processes with experimental plasticity models to explore the flexibility of spatiotemporal molecular mechanisms of disease has profound implications for patients with neurological disorders (71). In the future, the application of brain organoid bioengineering technology will greatly improve the vascularization of brain organoids, introducing the interaction of nerves and blood vessels, and prevent necrosis, to enhance physiological representation of the complexity of the human brain. Most current models do not effectively reflect the inflammatory response because they lack microglia. Therefore, an important addition to future models is the incorporation of immune cells and endothelial cells into brain organoids to fully understand how microbial fluctuations may regulate immune cell responses. In addition, multiple brain regions can be assembled into a complex system that ultimately reconstructs a complete brain-like organ that fully simulates a whole brain infection. Thus, highly regenerative brain organoids that better encapsulate the complexity of the human brain will be a powerful disease model with the capacity to comprehensively mimic brain disease, and will represent a revolutionary drug development strategy leading a new chapter in brain science research and personalized treatment.

## Author Contributions

FB, AL, and XS conceived and designed the study. PY, JK, and XX conducted the database search and screening. YZ, WC, and YF evaluated the data. ML, and JC conducted the quality assessment. XS drafted the manuscript. FB and AL revised and approved the manuscript. All authors contributed to the article and approved the submitted version.

## Funding

National Natural Science Foundation of China (32060180, 81860644, 81560596, and 31560051) and Joint Fundation of Yunnan Province Department of Science, Technology-Kunming Medical University [No. 2019FE001 (-002) and 2017FE467 (-001)] and and the Science Research Fund Project of Yunnan Provincial Department of Education (2021Y323). The funding institutions had no involvement in the design of the study or review of the manuscript.

## Conflict of Interest

The authors declare that the research was conducted in the absence of any commercial or financial relationships that could be construed as a potential conflict of interest.

## Publisher’s Note

All claims expressed in this article are solely those of the authors and do not necessarily represent those of their affiliated organizations, or those of the publisher, the editors and the reviewers. Any product that may be evaluated in this article, or claim that may be made by its manufacturer, is not guaranteed or endorsed by the publisher.
